# Evaluation of the implementation of a massive transfusion protocol

**DOI:** 10.1186/cc13300

**Published:** 2014-03-17

**Authors:** A Balvers, E Coppens, R Klinkspoor, AT Zeerleder, E Goslings, S Juffermans

**Affiliations:** 1 Academic Medical Center, Amsterdam, the Netherlands

## Introduction

A massive transfusion protocol (MTP) aims to provide standardized and early delivery of blood products and prohemostatic agents by keeping pre-thawed fresh frozen plasma (FFP) available. Implementation of MTP is assumed to result in transfusion with higher ratios of FFPs and platelets to red blood cells (RBCs). Pre-thawing may also result in waste of FFPs. These MTP benefits or disadvantages have not yet been demonstrated. The aim of this study was to evaluate efficacy of a MTP 1 year after implementation in our level I trauma center in an academic hospital.

## Methods

A retrospective analysis of an electronic blood bank transfusion database comparing massive transfusion before (January to December 2011) and after (January to December 2012) implementation of MTP. Activation of MTP consists of delivery of packages of 6 units of RBC, 6 units of pre-thawed FFP and 2 units of platelets collected from five donors. Massive bleeding was defined as transfusion ≥10 units of RBCs. Statistics by *t *test and Mann-Whitney *U *test.

## Results

In 2012, a total of 101 MTP activations was registered. Accurate prediction of massively bleeding patients (*n *= 30) was 29.7% of MTP activations. Of all massively bleeding patients in 2012, MTP was not activated in 55.2%. In patients for whom MTP was activated, the RBC:FFP ratio was 1:0.9. In patients for whom MTP was activated and who were massively bleeding, the RBC:FFP ratio was 1:0.9, which was significantly higher compared with 1:0.6 in massive bleeders in 2011 (*n *= 70) (*P *= 0.001). The median of blood products administered was 12.5 (6 to 21) in massive bleeding after MTP implementation, compared with 8 (3 to 13) in 2011 (*P *< 0.001). In patients for whom MTP was activated, 9.7% of thawed FFPs were not transfused and wasted. When massive transfusion was accurately predicted, the waste of FFPs was 4.8% versus a waste of 16.2% in the group of unjustified MTP activation.

## Conclusion

Implementation of MTP is associated with an increase of blood products transfused and a significant shift of RBC:FFP ratio to 1:1. However, MTP is also associated with a 9.7% waste of FFP. Improving prediction for massive bleeding patients may result in a decrease of wasted, costly blood products.

